# Proteomic Analysis in Search of New Biomarkers of Immune Thrombocytopenia (ITP)—A Review of Current Data

**DOI:** 10.3390/proteomes14010012

**Published:** 2026-03-12

**Authors:** Anastasia Boura-Theodorou, Konstantina Psatha, Stefania Maniatsi, Areti Kourti, Georgia Kaiafa, Michalis Aivaliotis, Kali Makedou

**Affiliations:** 1 Laboratory of Biochemistry, AHEPA General Hospital, School of Medicine, Faculty of Health Sciences, Aristotle University of Thessaloniki, GR-54124 Thessaloniki, Greece; ampourath@auth.gr; 2 Functional Proteomics and Systems Biology (FunPATh), Center for Interdisciplinary Research and Innovation (CIRI–AUTH), GR-57001 Thessaloniki, Greece; kpsatha@auth.gr (K.P.); maniatsi@auth.gr (S.M.); aivaliotis@auth.gr (M.A.); 3 Laboratory of Biological Chemistry, School of Medicine, Faculty of Health Sciences, Aristotle University of Thessaloniki, GR-54124 Thessaloniki, Greece; aretikourti@auth.gr; 4 Laboratory of Medical Biology—Genetics, School of Medicine, Faculty of Health Sciences, Aristotle University of Thessaloniki, GR-54124 Thessaloniki, Greece; 5 1st Propedeutic Internal Medicine Department, AHEPA General Hospital, School of Medicine, Faculty of Health Sciences, Aristotle University of Thessaloniki, GR-54124 Thessaloniki, Greece; gdkaiafa@auth.gr; 6 Basic and Translational Research Unit, Special Unit for Biomedical Research and Education, School of Medicine, Aristotle University of Thessaloniki, GR-54124 Thessaloniki, Greece

**Keywords:** immune thrombocytopenia (ITP), ITP pathogenesis, ITP diagnosis, proteomics, protein biomarkers

## Abstract

Immune thrombocytopenia (ITP) is a hematological disorder commonly found in individuals of any gender, race, or age. Patients with ITP will present with thrombocytopenia either in a primary form or because of an infection or a dysfunction in the immune system. The severity of ITP is linked to diminished production of platelets due to the blockage of production in the bone marrow niche and increased destruction of platelets, which confirms the diagnosis of the disorder. The investigation of the pathogenesis of ITP is of critical importance as it can give an important indication of the state of the patient, guiding us through risk assessment and treatment. Proteomics can provide tools to explore the protein profile of ITP. In this review, we aimed to uncover different biomarkers, both diagnostic and prognostic, that have been investigated with proteomic methodologies and that might help in understanding the pathogenesis of ITP and providing personalized treatment to patients. Several differentially abundant proteins were identified, including haptoglobin isoforms, heat shock proteins (HSPA6, HSPA8), integrin β3 (ITGB3), 14-3-3 protein eta (YWHAH), vitamin D-binding protein, fibrinogen chains, MYH9, and FETUB, which are involved in key signaling pathways, such as PI3K/akt, TNF-a, and mTOR, and they demonstrate potential as diagnostic and prognostic biomarkers. Collectively, current data support the value of proteomics for uncovering the molecular landscape of ITP and guiding the development of precision diagnostics and personalized therapeutic strategies.

## 1. Introduction

Immune thrombocytopenia (ITP) is an acquired autoimmune disorder portrayed as isolated thrombocytopenia, with a variable etiology and clinical manifestations [1,2]. Petechiae, purpura, and mucosal bleeding in the urinary tract, gastrointestinal, and oral cavities are the symptoms that appear in the diagnosis of ITP and are responsible for the patient’s poor prognosis and quality of life. In about 1% of the cases, patients confront fatal intracranial hemorrhages. Bleeding is detected in almost all symptomatic cases, but there seems to be a difference in the incidence of bleeding, and the platelet count does not differ. Also, 25–45% of patients with ITP report fatigue as one of the symptoms, which is independently associated with a platelet count of <30,000/μL, but not with treatment [3].

Under physiological conditions, ITP appears without any trigger and is recognized as primary immune thrombocytopenia (pITP). On the other hand, drug administration, underlying autoimmune and malignant disorders, like systemic lupus erythematosus (SLE), rheumatoid arthritis, antiphospholipid syndrome, Evan’s syndrome, common variable immunodeficiency, and chronic lymphocytic leukemia (CLL), as well as infectious diseases, such as human immunodeficiency virus (HIV), Epstein–Barr virus, cytomegalovirus, hepatitis B and C, and Helicobacter pylori, are set to promote secondary ITP (sITP) [4,5,6]. The classification of ITP does not rely solely on these underlying conditions. Three ITP categories have been introduced according to the persistence and timeline of the disease. The first one refers to the newly diagnosed ITP, as it involves the initial diagnosis and symptoms within three months. The second category is persistent ITP, where symptoms continue between 3 and 12 months from the diagnosis. ITP cases that exceed the symptomatic threshold of 12 months belong to the third category of chronic ITP [1]. The formerly known term “refractory ITP” was only attributed to patients with a failed attempt at a splenectomy and to those who are refractory to at least two prior therapies [4]. Nowadays, splenectomy is the least popular and efficient method of treatment, and consequently, the term “refractory” has been eliminated. Adult patients who have been diagnosed with pITP account for approximately 80% of the cases, and most of them progress to chronic ITP [1,5]. The remaining 20% of the patients present with sITP [6].

The pathogenic effect of platelet autoantibodies, ineffective thrombopoiesis (impaired maturation of megakaryocytes), and the excess destruction of platelets by T-cells are the leading causes of the disease [1,5]. It has been noted that while the platelet count is found excessively eliminated (<100,000/μL), given that the normal platelet count is 150,000 to 450,000/μL, white blood cell count (WBC) and hemoglobin levels retain their normal state [5]. ITP is commonly diagnosed in adults, adolescents, and children. The incidence in women is higher due to elevated estrogen, but this is reversed, especially after the age of 60 years [5]. Pediatric ITP is often persistent, is observed mostly after viral infections, and is self-limiting in 80% of cases [7]. ITP, even nowadays, remains a diagnosis of exclusion. No detectable underlying cause is usually found during investigation [8]. When it comes to the diagnosis of ITP in children, it is difficult to confirm due to the invasive nature of the procedure. Consequently, the entire diagnosis step is challenging and forestalls the initiation of treatment, with a consequent deterioration of the patient’s outcome. Examination of blood film is mandatory and may point to an alternative diagnosis, while bone marrow (BM) biopsy, according to the guidelines, is needed after unresponsiveness to first-line therapy or if the patients are older than 60 years.

According to the 2019 International Consensus Report on the investigation and management of ITP (ICR) [4] and the American Society of Hematology (ASH), the treatment of patients presenting with ITP should not rely solely on their staging but also on their needs. If the patient’s symptoms are excessive bleeding and a very low number of platelets, a first-line therapy is proposed, consisting of corticosteroids for newly diagnosed patients, as well as intravenous immunoglobulin (IVIG) and anti-D immunoglobulin. Immunosuppressive agents should be monitored, though, as they can promote the rise in autoimmune diseases. On the other hand, a second-line therapeutic strategy has been introduced for cases of persistent and chronic ITP, as well as for the relapsed patients. This kind of therapy involves newer and more targeted therapeutic agents that offer a more efficient prognostic value when compared to splenectomy, such as Thrombopoietin receptor agonists (TPO-RAs), rituximab, and fostamatinib [6,9,10,11]. The pathogenetic mechanisms that are involved in the emergence of ITP play a pivotal role in the advancement of innovative new treatments based on the inhibition of spleen tyrosine kinase inhibitors, Bruton’s tyrosine kinase (BTK), neonatal Fc receptors, or the complement pathway. The use of these new therapeutic agents alongside TPO-RAs is expected to improve OTP management [6].

An effort to better understand ITP and simultaneously facilitate the diagnosis has led to the investigation of new biomarkers, which can also serve as molecular fingerprints of ITP. Proteomics plays a pivotal role in the deciphering of hematological malignancies, as it is a state-of-the-art technique and a dynamic tool that can unravel the protein profile of a biological sample and provide innovative pathways to precision-medicine approaches as a new era of personalized treatment [12,13].

The scope of this review is to thoroughly present the existing literature regarding the proteomic profile of patients diagnosed with ITP and discuss the significance of discovering the molecular signatures of ITP for early diagnosis and new biomarkers for monitoring patients’ response to treatment. The ultimate goal is to present a comprehensive overview of the investigation of ITP pathogenesis through proteomic analysis and highlight the role of these high-end techniques in studying cellular pathophysiology and molecular mechanisms, and providing personalized treatment.

## 2. Materials and Methods

### Search Strategy and Selection Criteria

Our research was based on studies that were scavenged from platforms and biomedical databases such as PubMed, Elsevier, Scopus, Web of Science, and Frontiers. The keywords used in this study included: primary immune thrombocytopenia, immune thrombocytopenia (ITP), proteomics in the ITP diagnosis, pathogenesis of ITP studied through proteomic analysis, diagnostic tools of ITP using proteomics, and protein biomarkers of ITP. The inclusion criteria concerned studies on human samples and proteomic-based methodologies targeting diagnostic and prognostic biomarkers, whereas studies that involved animal testing, secondary ITP, ITP in association with other autoimmune diseases, ITP in pregnancy, and vaccine-induced ITP were excluded.

From the initial pool of 85 articles, only 10 met the inclusion criteria and were selected for this review (Table 1).

## 3. Pathophysiology of Immune Thrombocytopenia

Immune thrombocytopenia is an autoimmune disease that occurs because of the imbalanced interplay among effective and regulatory immune cells, which promotes ineffective thrombopoiesis and excessive destruction of platelets, mediated either by opsonization from antiplatelet antibodies or by T-cells [6]. The underlying pathogenetic mechanisms are quite intricate, as they involve multiple pathways, all of which can lead independently to the arousal of ITP.

The spleen is the main organ responsible for platelet homeostasis, being highly involved in the clearance of damaged or aged platelets from circulation, and macrophages that are present in the reticuloendothelial system are its major effector cells [6,23]. In the case of ITP, the production of antiplatelet antibodies, circulating in the blood, plays a pivotal role in the initiation of platelet clearance. IgG antiplatelet antibodies target platelet surface glycoproteins (GPs). The formation of circulating antibody-coated platelets activates the splenic reticuloendothelial system’s macrophages, which bind to the Fc region of immunoglobulins (IgGs) on platelets’ surface, via the FCγ receptor (FCγR) family. In immune thrombocytopenia pathogenesis, the splenic macrophages enable platelet phagocytosis through the Fc-FcgRIIa and Fc-FcgRIIIa interaction, with the involvement of spleen tyrosine kinase (Syk) [24]. Moreover, the major histocompatibility complex class II (MHCII) projects the generated phagocytosed platelet antigens to T-cell receptors (TCRs), which has a stimulatory effect on the autoreactive T-cells, like follicular T-cells (TFHs). The TFHs connect with autoreactive B cells through the CD40/CD154 axis and IL21 production, and consequently, B cells differentiate into autoreactive plasma cells, the production factory of antiplatelet antibodies. The platelet autoantibody overproduction results in the underproduction of platelets as a defect in the maturation of megakaryocytes.

In the active phase of ITP, the diminished establishment of regulatory T-cells (Treg) and their incapability of maintaining their self-tolerance lead to augmented platelet lysis, desialylation, and apoptosis, as well as to the overproduction of platelet autoantibodies [23,25]. In the case of platelet lysis, desialylation, and apoptosis, the low levels of Tregs fail to suppress the inhibition of megakaryopoiesis [26]. On the other hand, in the case of autoantibody overproduction, the insufficient number of Tregs reverses the suppression of B cell activation and differentiation into plasma cells, which, as mentioned before, will promote overproduction of antiplatelet antibodies [27].

Dendritic cells (DCs), the most influential antigen-presenting cells (APCs), can crosslink the innate and adaptive immune system. In ITP, DCs participate in apoptotic platelet phagocytosis and tend to activate the immunomodulatory cascade of autoantibodies by promoting the emergence of pathogenic T-cells. DCs are also responsible for the expression of co-stimulatory molecules CD86 and CD80, and for the intense production of IL-12, resulting in Treg insufficient self-tolerance and the immense destruction of platelets [6].

The research group of Li, J. et al. [24] succeeded in unraveling the implication of the liver in the platelet destruction in an FcγR-independent pathway, instrumented by anti-GPIbα antibodies, in contrast with the spleen. Under physiological conditions, hepatocytes and the spleen are responsible for platelet clearance. The latter is strongly associated with high serum levels of thrombopoietin (TPO), the substantial growth factor of the megakaryocytes produced by hepatocytes [6]. Since plasma coagulation factors are produced by the liver, the mechanism of action of anti-GPIbα antibodies promotes extensive complications in the coagulation cascade in ITP cases. Consequently, individuals presenting with thrombocytopenia face a severe bleeding phenotype. This research group observed that anti-GPIbα antibodies enabled platelet activation through an intracellular signaling cascade that resembles von Willebrand factor binding [24]. This finding led them to propose a novel antibody-promoted desialylation of GPIbα. Desialylation [28] is the gradual removal of sialic acid, which is abundant on the surface of platelets, and is mediated by sialidases [e.g., Neuraminidase 1 (NEU1) and Neuraminidase 3 (NEU3)]. This process takes place while platelets circulate in the peripheral blood (PB). In this research case, NEU1 was found to be the most implicated sialidase in the platelet clearance through desialylation and was introduced as a diagnostic tool and as a potential therapeutic pathway. Ashwell–Morell receptor (AMR), an asialoglycoprotein counter receptor produced by hepatocytes, has a pivotal connection with anti-GPIbα-mediated desialylation and platelet activation. AMR engages with senescent platelets, and after their desialylation, it initiates their FcγR-independent destruction [24,29].

## 4. Proteomics

Proteomics is a state-of-the-art suite of analytical techniques and a dynamic tool that enables protein characterization in a variety of biological systems, whether we refer to a cell, tissue, body fluid, or organism [30]. The main objective of proteomic analysis is protein identification, characterization, and localization, as well as identification of protein–protein interactions. The total amount of proteins that are present in a biological system under certain circumstances is the proteome [31]. The proteome is studied in favor of understanding the mechanism behind pathophysiological conditions because of its distinctive features and fluidity. Changes in the cell microenvironment or in organisms trigger the system’s cellular response, resulting in a different proteome scenery. The unraveling of protein biomarkers, which are implicated in a modified proteomic profile, with the use of proteomic analysis, may offer innovative pathways to precision-medicine approaches for a new era of specialized treatment [32].

Proteomic techniques involve, apart from protein quantitation and identification, interaction network analysis and post-translational modification (PTM) profiling [33,34]. The proteome’s diversity has been linked to different molecular processes, including alternative RNA splicing, genetic alterations such as mutations, and single-nucleotide polymorphisms (SNPs). Under these circumstances, proteoforms rise as structurally distinct conversions of genetically encoded proteins, displaying completely different tertiary structures and functional domains. Due to these alterations, proteoforms exhibit distinct protein–protein interactions and/or distinctive regulatory properties.

High-resolution, high-accuracy, and high-sensitivity MS platforms are key tools in protein and proteoform identification and characterization [35]. Top-down proteomics targets proteoform identification, characterization, and quantitation of intact proteins without tryptic digestion [36,37,38,39,40]. At the other end, in bottom-up proteomics, proteins are digested into peptides (typically <3 kDa) prior to nLC-ESI-MS/MS analysis. Bottom-up proteomics is by far the most used approach for protein sample characterization, as peptides are better analyzed by currently available technology [41]. Due to the large number of peptides that exist in studied protein samples, separation at a multifactorial level is often imperative and results in thorough examination of the proteome [42].

Different proteomics approaches can be used when analyzing peptides and reaching biomarker discovery, including MALDI-TOF MS/MS, SELDI-TOF MS, nanoLC-MS/MS Orbitrap, nano elute UHPLC MS/MS, and O-link proteomics. MALDI-TOF MS/MS is highly sensitive, simple, robust, and can support ionization and evaporation of large molecules, such as proteins, with high resolution due to the mass analyzer’s extremely large mass range in comparison to other mass analyzers [43,44]. However, it is a time-consuming and expensive process, and the steps for the preparation of the matrix often impair the quality of the spectra. On top of that, the results are rarely significant [43] SELDI-TOF MS follows the same principle as MALDI-TOF in terms of ionizing samples that have been co-crystallized with a matrix on a target surface with the use of a laser. It is a faster procedure that can run undigested biological samples, using a minuscule sample volume [43]. The disadvantages of this approach, while numerous, can be eliminated. For example, high-molecular-weight salivary proteins and glycoproteins, such as mucins, may not be visible. However, more mass peaks in the high-molecular-weight range could be acquired by applying different buffers or varying the stringency of the washing steps. Competitive binding of high-abundance non-informative proteins (like the mucins) on the chip surface could minimize the intensity of peptides/proteins of interest, particularly the low-abundance ones. The latter could be resolved by pre-fractionation of the saliva sample or the use of membranes with a specific cut-off. NanoLC-MS/MS, Orbitrap and nano-elute UHPLC MS/MS encompass liquid chromatography. The first one secures ultra-high sensitivity and resolution for complex samples, with Orbitrap’s superior mass accuracy resulting in high-quality characterization, often presenting the risk of a throughput downsize. On the other hand, nano-Elute UHPLC MS/MS offers high-throughput, robustness, and rapid analysis for large cohorts and a limitation in isomer separation [45]. Finally, while O-link proteomics relies solely on 1 μL of sample per panel, it is very limited on fixed pre-validated panels, its dynamic range is reserved for very low-abundance proteins, and a larger-scale process would be cost-effective [23]. The biggest limitations would be the ineffectiveness of directly measuring Post-Translational Modifications (PTMs).

## 5. Discussion—Discovery of Protein Biomarkers Through Proteomic Analysis

Immune thrombocytopenia is a challenging disease, and its treatment requires monitoring from multiple perspectives, which vary from the initial diagnostic features to selecting and establishing an invasive therapeutic approach. Those parameters led the way for exploring potential new biomarkers that could facilitate diagnosis, prognosis, and treatment with the help of proteomic analysis. There exist studies on subjects, humans, or animals that have or have not undergone treatment or splenectomy.

In an attempt to investigate the long-term response to splenectomy, Zheng et al. [14] studied the proteomic profile of serum in patients with pITP before and days after splenectomy. They used two-dimensional gel electrophoresis (2-DE) coupled with a MALDI-TOF/TOF-MS for protein identification and confirmed their results with the help of an enzyme-linked immunosorbent assay (ELISA). Their research managed to effectively introduce haptoglobin (Hp) and its isoforms (a-1, a-2, and β-chain of Hp) as potential serum biomarkers in responding and non-responding splenectomy patients. Hp is an acute-phase protein mainly produced in the liver by hepatocytes, and it is responsible for regulating the innate and adaptive immune mechanisms in response to several disorders. Hp is also capable of promoting B- and T-cell proliferation and functional differentiation, thus serving as an effector of homeostasis and a response to antigen-stimulatory pathways. In this study, Hp was established as a highly differentially abundant protein, and in particular, as preoperative Hp levels were high in responding patients and healthy controls, in contrast to low levels of Hp in non-responding patients. The latter correlated with the patient’s poor prognosis after surgery. On the other hand, preoperative Hp levels were not significantly different in patients and healthy controls. Lastly, preoperative serum levels of Hp were firmly linked to postoperative peak platelet count in ITP patients, and, consequently, to ITP pathogenesis, as Hp levels remain stable regardless of the circumstances and the patient’s status.

Zhang et al. [15] aimed to propose a more clinically applicable diagnostic approach, which could potentially enable treatment of ITP. They collected 134 samples of platelet plasma from patients with pITP and sITP and evaluated their proteomic profile using SELDI-TOF-MS analysis. Proteomic analysis was followed by artificial neural networks (ANN), with the purpose of introducing a diagnostic model, using the following results. A successful identification of 96 proteins in total was established. Fifteen proteins were differentially abundant among pITP and sITP patients, and after statistical analysis, five proteins were found to be significantly up- or down-regulated. Cortistatin (CORT), the only protein found down-regulated, is a sleep-modulating neuropeptide that is found in human central and peripheral tissues after triggering human immune cells. Neuropeptide S (NPS) is an arousal-promoting peptide that was up-regulated in platelet plasma of patients with pITP and is abundant in cases with increased pathogen phagocytosis performance. NPS secretion has been found linked to macrophage stimulation and thus to phagocytosis. Moving forward, during the analysis of MS spectra, they observed two potential markers in the same peak area: endothelin-1 (EDN1) or natriuretic peptide B (NPPB), which were up-regulated. EDN1 is produced by macrophages, whereas NPPB is released by ventricles of the heart and is associated with the pathogenetic mechanisms and prognostic features of cardiac diseases. Another up-regulated, identified marker for pITP was C-type lectin domain family 7 member A (CLEC7A), a member of the C-type lectin/C-type lectin-like domain (CTL/CTLD) superfamily. The latter represents a C-type lectin-like protein (CLP). This set of proteins is involved in platelet activation and is also responsible for initiating the immune defense response against pathogens. Chemokine (C–C motif) ligand 18 (CCL18) was the fifth up-regulated identified marker. CCL18 has proven to be an additional effector of macrophage activation, and, in combination with all the above-identified markers, a potential immunity-related pathogenetic target for pITP treatment options.

**Figure 1:**
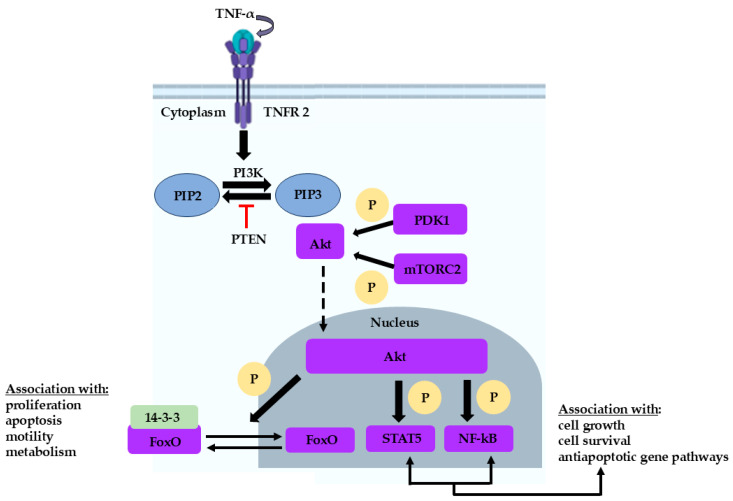
Schematic representation of the PI3K/Akt pathway. When TNF receptor 2 (TNFR2) is activated, the intracellular domains recruit existing cytoplasmic complexes, resulting in the activation of the PI3K/Akt pathway. On top of that, activation of Akt signaling through phosphoinositide-dependent protein kinase (PDK1) and mammalian target of rapamycin (mTOR) complex 2 (mTORC2) allows the interaction of the PI3K/Akt pathway with STAT5 and NF-kB (increased phosphorylation). Consequently, the PI3K/Akt pathway can indirectly control cellular functions. Akt also phosphorylates and inactivates forkhead box O (FOXO) transcription factors in the nucleus, thereby dampening the expression of FOXO target genes involved in proliferation, apoptosis, motility, and metabolism (Figure originally created by the authors).

In the published research of Liu Sy et al. [16], the BM of newly diagnosed patients with ITP versus controls was studied using high-pH HPLC and LC-MS/MS analysis, with the scope of discovering a potential implication of apoptosis in ITP pathogenesis. They focused on five proteins that were found down-regulated: HSPA6, HSPA8, YWHAH, ITGB3, and PRDX6. HSPA6 and HSPA8 are members of the heat shock family of 70 kDa proteins. Under physiological conditions, heat shock proteins of 70 kDa are responsible for inhibiting protein-promoters of the apoptotic signaling pathways, such as caspases. A down-regulation of these proteins results in enhanced apoptosis. Enrichment analysis of YWHAH showed a multifactorial implication of the protein in ITP pathogenesis when down-regulated. Further elaborating, YWHAH abundance is connected to mitotic cell death, which will mediate cell death via apoptosis, as well as to the Hippo signaling pathway, which plays a crucial role in balancing cell proliferation and apoptosis. Negative regulation of YWHAH induces the PI3K-Akt pathway (Figure 1) and results in negative regulation of apoptosis. ITGB3, on the other hand, is involved in platelet activation and aggregation. When ITGB3 is mutated, platelet dysfunction and macrothrombocytopenia will be observed. The latter derives from the formation and activation of integrin alpha IIb/beta 3 (α_IIb_β_3_) and is initiated via the PI3K-Akt pathway. That is because α_IIb_β_3_ acts as an effector of blood clot contraction and thrombosis. ITGB3 thrives under hypoxic conditions, and when it is dysregulated, it negatively affects survival and migration with the emergence of apoptotic signals. The protein markers proposed in this study were found in abundance in the thioredoxin domain, a part linked to PRDX6, a peroxidase capable of regulating cell proliferation, while also acting as a shield against oxidative stress with the help of PI3K-Akt pathway activation. When PRDX6 is down-regulated, ROS levels increase, causing oxidative stress and apoptosis. This study provided helpful information on the implications of apoptosis via the PI3K-Akt pathway that can enhance the introduction of new treatment strategies for ITP.

Sun RJ et al. [17] isolated Bone Marrow Mesenchymal Cells (BMMCs) from BM aspirates of newly diagnosed patients with pITP and healthy controls. Their study group aimed to find a connection between autophagy and ITP. Autophagy is a preserved catabolic process that controls the function of the innate immune system, the emergence of tumors, and cell development. Dysfunction in maintaining this intracellular quality check, as well as the stemness of the hematopoietic stem cells, along with the cell’s microenvironment, leads to defects in megakaryopoiesis, diminished levels of proplatelets, and ultimately reduced platelet formation and function. To prove their theory on the potential connection of autophagy and ITP, they performed high-pH RP-HPLC and tandem LC MS/MS, followed by enrichment analysis and parallel reaction monitoring analysis, aiming to verify the implication of the identified protein markers. A total of 26 of the quantified proteins were up-regulated and linked to the adaptive immune system, whereas 69 down-regulated proteins were implicated in autophagy. Five of those were found differentially abundant and related to autophagy. CSF1R was the only up-regulated protein in the BMMCs of patients versus the control group. HSPA8, YWHAH, ITGB3, and PARK7 were the other four differentially abundant proteins and were shown to be down-regulated in the BMMCs of patients with ITP when compared to healthy controls. CSF1R, YWHAH, and ITGB3 share a connection to the PI3K/Akt/mTOR signaling pathway. YWHAH, apart from its role in apoptosis, belongs to a large group of phosphoregulatory proteins that are significant in modulating cellular and whole-body energy. On top of that, YWHAH is associated with the nutrient homeostatic mechanisms of insulin and mTOR- and AMP-dependent kinase (AMPK) tracks to control autophagy. AMPK promotes the inhibition of the regulatory-associated protein of TOR (RAPTOR) via phosphorylation-induced binding of YWHAH, controlling autophagy initiation. Under physiological conditions, ITGB3 plays a critical role in cell survival, proliferation, and cancer metastasis, and is involved in the Akt activation process when up-regulated, restraining cell autophagy. Low levels of ITGB3 result in diminished activation of Akt and, consequently, in autophagy-related emergence of ITP. CSF1R was found to be down-regulated in the control group versus the patient study group. This protein is strongly associated with the differentiation of human monocytes into macrophages, as it induces autophagy-related molecules, like phagophores and autophagosomes, so CSF1R’s differential abundance could induce abnormal autophagic reactions leading to ITP pathogenesis. HSPA8 abundance, as shown above, is linked to the induction of apoptosis. This study contributed to a better understanding of its function as a molecular chaperone, as well as its role in the arousal of immune disorders such as ITP, because HSPA8 can detect substrates that have undergone changes by chaperone-mediated autophagy. These substrate alterations cause a modification of HSAP8 abundance, making it eligible to be used as a marker of autoimmunity. Finally, PARK7 is an antioxidant protein responsible for supporting mitochondrial homeostasis and cell viability. Consequently, down-regulation of PARK7 in patients with ITP may increase cell vulnerability.

Gilanchi S. et al. [18] combined high-throughput approaches and computational methods with the scope of proposing new plasma biomarkers that could serve as first-line therapy effectors. They used PB plasma of newly diagnosed patients with pITP and chronic ITP, before and after treatment, divided them into responders and non-responders, and compared it to that of healthy controls. 2-DE was performed for the localization of differentially abundant proteins, followed by protein identification via MALDI-TOF MS analysis. Nineteen plasma proteins were identified as differentially abundant, one of which was up-regulated, and the remaining eighteen were down-regulated. The SwissProt database’s extracted data revealed sixteen differentially abundant proteins. Protein–protein interaction network analysis introduced three distinctly abundant proteins, APOA1, TRFE, and VITDB, that were important indicators of a patient’s therapy response. The latter was verified through the method of sandwich ELISA. All three proteins were found in low levels in patients with a negative response to therapy. Since these markers participate in platelet degranulation, transporting and storing iron and vitamin D, changes in their abundance could be applied in the treatment. FIBB, FIBG, and SCRIB were also identified as protein biomarkers for monitoring treatment, as they play a crucial role in platelet activation and apoptosis, which are involved in ITP pathogenesis and could be targeted in treatment strategies.

Cao Q. et al. [10] were determined to explore plasma protein biomarkers in children with ITP. Diving into detail, patients presenting with ITP may be resistant to glucocorticoid therapy, which is a common and, usually, effective way of treating ITP, leading, however, to a poor quality of life due to the appearance or deterioration of symptoms and delay in the disease’s remission. In order to facilitate the treatment formulation for ITP and to avoid the side effects of delaying targeted therapy, the researchers employed a four-dimensional (4D)-DIA proteomics methodology and aimed to discover differentially abundant proteins in pediatric patients with pITP under glucocorticoid treatment, and they validated the results using ELISA. They collected 35 PB plasma samples and divided the patients according to their response to the drug into glucocorticoid-sensitive (GCS) and glucocorticoid-resistant (GCR) groups. They also included 30 healthy controls. A total of 47 differentially abundant proteins were identified, of which 36 were up-regulated and 11 were down-regulated in comparison with the GCS patients. The altered protein abundance between the GCS and GCR groups of patients was significant. Among the identified proteins, two were proposed as potential biomarkers for the glucocorticoid efficacy in treating pediatric ITP cases according to their *p* values. MYH9 was found significantly down-regulated in GCR patients and abundant in patients versus healthy controls. This cytoskeletal protein has been introduced as an effector in a variety of cellular pathways, like migration and signaling, and is also involved in the regulation of the hematopoietic system. MYH9 gene mutations affect hematopoiesis and result in thrombocytopenia-related diseases. FETUB, on the other hand, is a liver-derived protein and was found up-regulated in GCR patients when compared to the GCS group, whereas its patient levels were diminished in contrast to controls. This protein shares structural similarities with FETUA, an endogenous protein that participates in the initiation of inflammation when bound to TLR. The TLR activation-related pathway projects FETUB as a potential biomarker for establishing glucocorticoids as effective ITP treatment, as the inflammatory response that derives from FETUB’s differential abundance is a promoter for glucocorticoid resistance.

Yin Dm. et al. [19] focused on revealing the mechanism behind the loss of immune tolerance in cases of pITP patients, with the help of quantitative liquid chromatography tandem mass spectrometry (LC-MS/MS) analysis. For the purposes of this study, they isolated BMMCs from newly diagnosed patients and healthy controls. A total of 829 identified proteins were found to be differentially abundant. From the total number of proteins, 26 were up-regulated and 69 were down-regulated. Four of these proteins were significantly altered and connected to immune deregulation and to ITP pathogenesis. ORM1 and vWF were up-regulated, whereas PPBP and SPARC were down-regulated. The results of this study demonstrated a strong interaction between these proteins, and more specifically, their involvement in the TNF-α signaling pathway, and, consequently, in the emergence of autoimmune diseases, such as ITP. ORM1 is a cytokine-regulated acute-phase protein observed in high abundance in inflammatory sites. The up-regulation of ORM1 in ITP patients leads to an increased abundance of TNF-α or activation of TNF-α’s mediators (NF-κB, p38 and JNK, and MAPK signaling), which results in lower platelet number and in thrombocytopenia. vWF glycoprotein participates in thrombosis and hemostasis and stands as an effector of platelet adhesion to subendothelial tissues. It also participates in inflammation with the same pattern as ORM1, through the TNF-α signaling pathway. The escalation of TNF-α levels promotes vWF production from endothelial cells and triggers the immune system. Moreover, TNF-α can negatively affect the regulation of SPARC, a mediator of cell proliferation and angiogenesis, and a promoter of tissue fibrosis, leading to an inflammation outburst. PPBP, which is also known as CXC chemokine ligand 7 (CXCL7), participates in neutrophil production during thrombosis, and so a dysfunction in PPBP can cause the activation of inflammatory signaling pathways. The latter is enhanced by the modulatory role of TNF-α as well. Their findings led them to formulate a hypothesis, where all the differentially abundant identified proteins join forces and activate the TNF-α signaling pathway, which is directly linked to ITP pathogenesis.

Xu W. et al. [20] focused on pediatric cases with thrombocytopenia. Children often refuse to undergo bone marrow aspiration, which favors a poor prognosis due to a lack of immediate treatment planning. This study group investigated PB plasma samples from patients with pITP and secondary ITP, and healthy controls. They used 4D-DIA proteomics, a nano-LC-MS/MS system specifically, for the detection of 1586 proteins, with 43 found in high abundance and 12 in low abundance. Two proteins were found to be differentially abundant and were proposed as potential diagnostic biomarkers. MMP-9 is a gelatinase that was found to be up-regulated in plasma samples. The up-regulation of enzymes like MMP-9 is associated with ITP pathogenesis because they favor TGF-β pathway signaling and deregulation of the red blood system. The latter is reversible due to the MMP-9 inhibitors. THBS1, on the other hand, was found to be down-regulated, which directly affects platelet activation. THBS1 is involved in angiogenesis, aggregation, and tumor development, and it can suppress the effect that adenosine monophosphate signaling has, as it tends to negatively regulate those biological processes. Overall, the significant alterations observed in these proteins led to the suggestion for their selection as ITP-related diagnostic biomarkers.

The discovery of diagnostic protein biomarkers for immune thrombocytopenia was also the aim of the study of Jiang Y. et al. [21]. This research group focused on adult ITP and enrolled PB plasma samples of patients with pIPT and sITP and healthy controls. Their approach involved O-link proteomics as a new methodology of protein identification via high-throughput screening of protein biomarkers. Twenty-two identified proteins were differentially abundant and were directly linked to ITP pathogenesis and, consequently, were introduced as diagnostic and/or prognostic markers for adult ITP, which nonetheless needs further validation.

To shed light on the pathophysiology of ITP and potentially enhance treatment advancement, Li J. et al. [22] performed a label-free quantitative proteomic analysis on BMMCs and exosomes isolated from BM plasma samples of patients with ITP and healthy donors. They identified three proteins that were differentially abundant and in high abundance in PB plasma samples when compared to healthy donors. MBL2, FCN2, and CFP are hepatocyte-derived proteins that promote platelet production via thrombopoietin activation, and their deregulation leads to dysfunction in the activation of the lectin complement pathway and in increased platelet elimination. Their results offer insights into the role of exosomes in the regulation of immune systems and their involvement in the ITP pathogenetic profile, as well as in a variety of autoimmune diseases.

## 6. Conclusions

Immune thrombocytopenia is an autoimmune disease with varying etiology and clinical symptoms. Its pathogenetic nature revolves around the imbalance between the effective, regulatory immune system and apoptotic and autophagic pathways that promote increased platelet destruction. The versatility behind the different pathways of ITP triggering renders its treatment a challenging procedure and is indicative of poor prognosis. Also, due to the invasive form of the diagnostic means necessary, this process may hinder its immediate treatment options.

Proteomics is a state-of-the-art suite of methodologies capable of identifying proteins and investigating in-depth their modification state. MS analysis provides a high-throughput library of global proteomic profiles that serves as a pool of new protein biomarkers that can enable treatment strategy formation and even be introduced as a diagnostic and/or prognostic tool. The existing literature regarding proteomic profiling of ITP has helped with the emergence of new biomarkers, but most of the studies require further validation in a clinical setting.

## Figures and Tables

**Table 1:** Overview of research articles and emerging biomarkers discussed in the review (ITP: Immune thrombocytopenia; BM: Bone marrow; BMMCs: Bone marrow mononuclear cells; PBMCs: Peripheral blood mononuclear cells, upwards red arrows: increased abundance in sample, downwards red arrows: reduced abundance in samples).

Authors	Year	Sample Type	Sample Number	Proteomics Approach	Potential Biomarkers	Biomarker Trend	Association with ITP
Zheng CX. et al. [14]	2012	Blood serum	58	2-DE MALDI-TOF/TOF MS	a-1, a-2 isoforms of haptoglobin β chain of haptoglobin	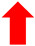	ITP pathogenesis
Zhang HW. et al. [15]	2016	Platelet plasma	134	SELDI-TOF MS	Cortistatin (CORT) Neuropeptide S (NPS) C-type lectin domain family 7 member A (CLEC7A) Chemokine ligand 18 (CCL18) Endothelin-1 (EDN1)/ Natriuretic peptide B (NPPB)	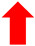	Immunity-related ITP pathogenesis
Liu Sy. et al. [16]	2021	BM serum	20	Nano-LC-MS/MS Orbitrap MS	Heat shock 70 kDa protein 6 (HSPA6) Heat shock cognate 71 kDa protein (HSPA8) 14-3-3 protein eta or η (YWHAH) Integrin beta-3 (ITGB3) Peroxiredoxin-6 (PRDX6) Heat shock protein 70 (HSP70)	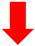	Apoptosis in ITP and pathogenesis through PI3K-Akt pathway
Sun RJ. et al. [17]	2021	BMMCs	40	LC-MS/MS Orbitrap MS	Colony-stimulating factor 1 receptor (CSF1R) Heat shock cognate 71 kDa protein (HSPA8) Parkinson’s disease 7 (PARK7) Tyrosine 3-monooxygenase/tryptophan 5-monooxygenase activation protein eta polypeptide isoform CRA_b (YWHAH) Integrin beta-3 (ITGB3)	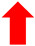 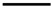 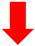	Autophagy-related ITP pathogenesis
Gilanchi S. et al. [18]	2023	Blood plasma	16	2-DE MALDI-TOF MS	Vitamin D-binding protein (VTDB) Serum iron transport protein ransferrin (TRFE) Apolipoprotein A-1 (APOA1) Scribble planar cell polarity protein (SCRIB) Fibrinogen beta chain (FIBB) Fibrinogen gamma chain (FIBG)	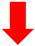	ITP therapy monitoring
Cao Q. et al. [10]	2023	Blood plasma	65	NanoElute UHPLC-MS/MS	Heavy chain myosin-9 (MYH9) Fetuin B (FETUB)	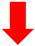 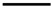 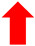	Glucocorticoids in ITP treatment and ITP pathogenesis
Yin Dm. et al. [19]	2023	BM serum, BMMCs	40	LC-MS/MS	Orosomucoid 1 (ORM1) Von Willebrand factor (vWF) Pro-platelet basic protein (PPBP) Secreted protein acidic and rich in cysteine (SPARC)	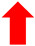 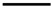 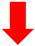	ITP pathogenesis
Xu W. and Wang Y. et al. [20]	2024	Blood plasma	40	4D-DIA quantitative proteomics Nano-LC-MS/MS	Matrix metalloproteinase 9 (MMP-9) Thrombospondin-1 (THBS1)	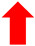 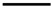 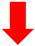	ITP pathogenesis and diagnosis
Jiang Y et al. [21]	2025	Blood plasma	92	Olink proteomics	Angiopoietin 1 (ANGPT1) Platelet-derived growth factor (PDGF) subunit B T-cell surface glycoprotein CD40 ligand (CD40-L) C-X-C motif chemokine 11 (CXCL11) Latency-associated peptide-transforming growth factor-beta 1 (LAP TGF-beta-1) Tumor necrosis factor (ligand) superfamily, member 14 (TNFSF14) C-X-C motif chemokine 12 (CXCL12) Interleukin-4 (IL4)-transforming growth factor beta-1 proprotein (TGF-β1) Tumor necrosis factor (TNF) Arginase-1 (ARG1) T-cell surface glycoprotein CD5 (CD5) Matrilysin (MMP7) C-C motif chemokine 2 (CCL2) Granzyme H (GZMH) Nitric oxide synthase, endothelial (NOS3) Interleukin-13 (IL13) C-C motif chemokine 8 (CCL8) C-C motif chemokine 13 (CCL13)	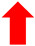	ITP diagnosis and treatment
Li J. et al. [22]	2025	BMMCs, PBMCs, BM plasma exosomes	9	Q Exactive HFX Hybrid Quadrupole-Orbitrap MS Easy nLC	Exosomal mannose-binding lectin 2 (MBL2) Exosomal ficolin 2 (FCN2) Exosomal complement factor properdin (CFP)	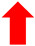	ITP pathogenesis

## Data Availability

There was no new data.
